# Ten influenza seasons in France: distribution and timing of influenza A and B circulation, 2003–2013

**DOI:** 10.1186/s12879-015-1056-z

**Published:** 2015-08-21

**Authors:** Anne Mosnier, Saverio Caini, Isabelle Daviaud, Jean-Louis Bensoussan, Françoise Stoll-Keller, Tan Tai Bui, Bruno Lina, Sylvie Van der Werf, Jean Marie Cohen, on behalf of the GROG network

**Affiliations:** Open Rome (Organize and Promote Epidemiological Network), 67 rue du Poteau, 75018 Paris, France; Réseau des GROG, 67 rue du Poteau, 75018 Paris, France; Laboratoire de Virologie, CHU de Strasbourg, Strasbourg, France; CNR des virus influenzae, CBPE, HCL & Virpath, UCBL, Université de Lyon, Lyon, France; CNR des virus influenzae, GMVR Institut Pasteur, CNRS UMR3569, Université Paris Diderot, Sorbonne Paris Cité, Paris, France

**Keywords:** Influenza surveillance, Influenza virus type A, Influenza virus type B, Influenza vaccine match, Influenza incidence, Timing of influenza epidemics

## Abstract

**Background:**

Describing the circulation of influenza viruses and the characteristics of seasonal epidemics remains an essential tool to optimize the strategies of influenza prevention and control. Special attention has been recently paid to influenza B in the context of the availability of a quadrivalent vaccine, containing two influenza B strains.

**Methods:**

We used data from a practitioners-based influenza surveillance network to describe the circulation of influenza viruses in France from 2003–2004 to 2012–2013. Nasopharyngeal swabs taken from acute respiratory infection (ARI) patients between October and April were tested for influenza. We reported the number of influenza cases by virus type (A, B), subtype (A(H1), A(H3)) and B lineage (Yamagata, Victoria) in each season and determined the frequency of influenza B vaccine mismatch. We estimated weekly incidence of influenza by extrapolating reported influenza cases to the French population. We compared the temporal characteristics of the epidemics caused by influenza A(H1), A(H3) and B.

**Results:**

Overall, 49,919 ARI patients were tested, of which 16,287 (32.6 %) were positive for influenza. Type B virus caused 23.7 % of all influenza cases. Virus subtypes A(H1) and A(H3) caused 51.6 % and 48.4 % of influenza A cases, respectively. Viruses of the B-Yamagata and B-Victoria lineage caused 62.8 % and 37.2 % of influenza B cases, respectively. There was an influenza B vaccine mismatch in three of the five seasons where influenza B caused 10 % or more of all influenza cases. Influenza A(H3) had the highest average value of estimated weekly incidence during the study period. Influenza B peaked an average 3.8 weeks later than influenza A when both virus types were circulating. No differences in the duration of influenza A and B epidemics were observed.

**Conclusions:**

Influenza A(H3) was the most prevalent influenza type during the study period. Influenza B caused around one fourth of all influenza cases and tended to circulate later than influenza A. The frequency of influenza B vaccine mismatches was substantial. Timely data on the circulation of influenza viruses collected within influenza surveillance systems are essential to optimize influenza prevention and control strategies.

**Electronic supplementary material:**

The online version of this article (doi:10.1186/s12879-015-1056-z) contains supplementary material, which is available to authorized users.

## Background

Until a few years ago, inactivated vaccines against influenza were only available in France as a trivalent formulation, i.e. containing hemagglutinin of two strains of type A influenza virus (A[H3N2] and A[H1N1]) and of only one strain of type B influenza virus, belonging to either the B-Yamagata or B-Victoria lineage [1]. Type B influenza has evolved into two distinct lineages, Yamagata and Victoria, from which strains of both have been co-circulating since 2001 [2]. Recently, quadrivalent vaccines with a B strain from each lineage have been developed to reduce lineage mismatch of influenza vaccine. Quadrivalent vaccines have been added to the WHO recommendation in Northern hemisphere starting the season 2013–2014 [3, 4]. It is expected that this will result in an improved performance of influenza vaccination campaigns in terms of both public health and economic outcomes [5, 6].

Influenza variability, as exemplified above, calls for a detailed and continuous description of virus circulation and characteristics of seasonal epidemics. Comprehensive surveillance in general population that integrates clinical and virological data (including laboratory testing, strain typing and phylogenetic analysis of circulating viruses) remains a very useful tool to optimize any strategy for influenza prevention and control, and is a prerequisite to calculate vaccine effectiveness through observational studies.

Here, we aimed to provide a descriptive analysis of circulating influenza viruses in France during ten consecutive seasons (from 2003–2004 to 2012–2013) using data from a nationwide community-based sentinel influenza surveillance network. Results are provided on the distribution of specimens testing positive for influenza by virus type (A, B), subtype (A(H1), A(H3)) and lineage (B-Yamagata, B-Victoria) in each season; on the match between the influenza B lineage included in the vaccine and the dominant circulating B-lineage viruses; on the estimated medically-attended influenza incidence by virus type and subtype; and on the timing of influenza A vs. B epidemics.

## Methods

This is a retrospective descriptive study including data collected during ten consecutive seasons (from 2003–2004 to 2012–2013) within the GROG (Groupes Régionaux d’Observation de la Grippe) influenza surveillance network in France.

### The GROG network

The GROG is a French countrywide influenza sentinel surveillance network based on voluntary primary care physicians (general practitioners [GPs] and paediatricians), covering 21 of 22 French regions [7–9]. The GROG was established in 1984 and has been collecting and linking clinical, virological and demographic data since then. Active surveillance of influenza runs every season from week 40 to week 15.

The number of physicians participating to the GROG network has been constantly increasing since its establishment: in 2012–2013, 0.64 % of general practitioners and 4.44 % of paediatricians in France participated in the GROG network, with a high participation rate (73 % of general practitioners and 76 % of paediatricians swabbed at least one person per season), and with a distribution on the French territory representative of the general population of French GPs and paediatricians. Characteristics of participating practitioners (like age and gender distribution) do not differ from those of non-sentinel practitioners throughout France.

### Selection of patients and data collection

The sentinel physicians participating in the GROG network reported the weekly number of acute respiratory infections (ARI) patients presenting at their practice, and collected information and provided, on a judgmental sampling basis, nasal/pharyngeal swabs from a subset of ARI patients presenting within 48 h of onset of symptoms. The definition of ARI adopted was as follows: sudden onset of at least one respiratory sign (cough, sore throat, shortness of breath, coryza…) AND at least one systemic sign suggestive of an acute infectious disease (fever, fatigue, headache, malaise…). The information being collected included socio‐demographic data, clinical symptoms, and whether the patient was vaccinated in the current and (from season 2009–2010) previous season.

The data collected from ARI patients that were swabbed and the results of the virological analyses were entered into a protected database named “Vircases” located at the Open Rome headquarters, Paris, France.

### Sampling procedures and laboratory diagnosis

Sentinel practitioners took nose or throat swabs from some of the patients that met the definition of ARI in use within the GROG network. Specimens were transported to the laboratory by post (together with the routine surveillance clinical form), with a triple packaging system following the international guidelines for the transport of infectious substances (category B, classification UN 3373).

The virological analyses were performed in one of the two French National Influenza Centers (NIC) in Paris and Lyon or in one of the regional laboratories (six to eight depending on the season) collaborating with the GROG network. The specimens of swabbed ARI patients were analyzed for the presence of influenza viruses and in most cases, if influenza positive, the virus type and subtype (for influenza A cases) were determined. Until 2008–2009, laboratory confirmation essentially relied on enzyme immunoassays for determination of virus type, and isolation in cell culture followed by hemagglutination inhibition assays using specific polyclonal sera for determination of virus sub-type and antigenic characterization. Since 2009–2010, all the laboratories have been mainly performing real-time reverse transcriptase polymerase chain reaction (RT-PCR) tests for virus detection, (sub)-typing and determination of influenza B lineage [10]. According to the terms of reference of the National Influenza Centers and WHO collaborating centers, laboratory procedures for strain typing were validated at the WHO Collaborating Centre for Reference and Research on Influenza of the National Institute for Medical Research at Hill Mill, UK, using a ten percent sample. Following the WHO collaborating centers recommendations, the lineage of influenza B viruses was characterized only in a random subset of specimens that were sent to the NIC [11].

### Statistical analysis

ARI patients diagnosed during the European influenza surveillance seasons 2003–2004 to 2012–2013 (that is to say between weeks 40 and 15, October to April) were included in the present analysis. We excluded from the analyses the ARI patients that were concomitantly positive for both influenza A and B or for two (or more) influenza A subtypes. For each season, we reported the number of samples from ARI patients that were tested, the percentage of influenza positivity, and the number of influenza cases by virus type (A, B), subtype (A(H1), A(H3)) and, when influenza B accounted for at least 10 % of all influenza cases detected during the season, by lineage (B-Yamagata, B-Victoria) as well. In the 2009–10 season, influenza A(H1N1) pdm09 strain completely replaced previously circulating A(H1N1), but both are referred to as A(H1) in this paper. For seasons with at least 10 % of all influenza cases due to influenza type B virus, we determined whether there was a mismatch between the dominant B lineage (>60 % of B isolates as defined by ECDC) and the lineage included in the vaccine composition [3].

The weekly incidence of medically-attended influenza was estimated using the method developed within the GROG network, which is available as Additional file 1.

The temporal characteristics of the epidemics caused by influenza viruses A(H1), A(H3) and B were examined when they accounted for at least 10 % of all influenza cases that occurred during the whole season. After defining the peak of influenza activity as the week with the highest reported number of isolates belonging to that virus type/subtype, we calculated, for each virus type and subtype, the duration (in weeks) of the shortest period that (1) included the peak (but was not necessarily centred on it), and (2) during which at least 90 % of all influenza cases that were caused by that virus type/subtype during the whole season were diagnosed. Whenever two or more periods matched this definition, we chose that with the highest percentage of influenza cases due to that virus type/subtype. This period can be regarded as that of the most active circulation of each influenza virus type and subtype in the population in each given season.

### Ethical aspects and informed consent

Surveillance forms were routinely used in the influenza seasons, and oral informed consent was obtained from the ARI patient at the moment of swab taking in accordance with national regulations. All swab results and forms were anonymized by the laboratories before they were sent to the GROG network coordination, and only identified by the number given by each laboratory for virological tests. In accordance with applicable laws and regulations, no clearance of an Ethics Committee is required in France for the retrospective analysis of anonymized data collected within routine influenza surveillance schemes.

## Results

An average 530 general practitioners and paediatricians participated every season in the GROG network (range: 498–608), of which an average 466 swabbed at least one ARI patient each season (range: 403–578).

Over the whole study period, 49,919 naso-pharyngeal specimens were collected and tested for influenza viruses (Table 1), which corresponds to around 3 % of all ARI patients seen by participating practitioners. The number of specimens that were collected each season ranged between 2,737 in 2003–2004 and 5,447 in 2012–2013 for seasonal influenza; it was 8,822 in the pandemic season 2009–2010. The total number of specimens that tested positive for influenza viruses was 16,312, of which 16,287 were used for the analysis (Table 1). The number of influenza cases reported per season ranged between 903 in 2005–2006 and 2,613 in 2012–2013; it was 3,105 in 2009–2010. The overall proportion of specimens that tested positive was 32.6 %, ranging between 18.6 % in 2005–2006 and 48.0 % in 2012–2013. The proportion of influenza virus positive samples that belonged to influenza type B ranged between <1 % (in 2003–2004, 2006–2007 and 2009–2010) and 62.3 % in 2005–2006. Overall, 23.7 % of influenza positive swabs belonged to type B from 2003–2004 to 2012–2013.

**Table 1:** Number of specimens tested and influenza cases, and percentages attributable to influenza A and B virus types

Season	Specimens tested	Influenza cases	% positive	A	B
2003–2004	2,737	950	34.7 %	946 (99.6 %)	4 (0.4 %)
2004–2005	3,782	1,118	29.6 %	1,018 (91.1 %)	100 (8.9 %)
2005–2006	4,858	903	18.6 %	340 (37.7 %)	563 (62.3 %)
2006–2007	4,609	1,061	23.0 %	1,055 (99.4 %)	6 (0.6 %)
2007–2008	5,084	1,375	27.0 %	893 (64.9 %)	482 (35.1 %)
2008–2009	4,860	1,564	32.2 %	1,336 (85.4 %)	228 (14.6 %)
2009–2010	8,822	3,105	35.2 %	3,098 (99.8 %)	7 (0.2 %)
2010–2011	5,255	2,068	39.4 %	1,086 (52.5 %)	982 (47.5 %)
2011–2012	4,465	1,530	34.3 %	1,474 (96.3 %)	56 (3.7 %)
2012–2013	5,447	2,613	48.0 %	1,189 (45.5 %)	1,424 (54.5 %)
Total	49,919	16,287	32.6 %	12435 (76.3 %)	3852 (23.7 %)

Source: GROG influenza sentinel surveillance network, France, 2003–2004 to 2012–2013

**Fig. 1:**
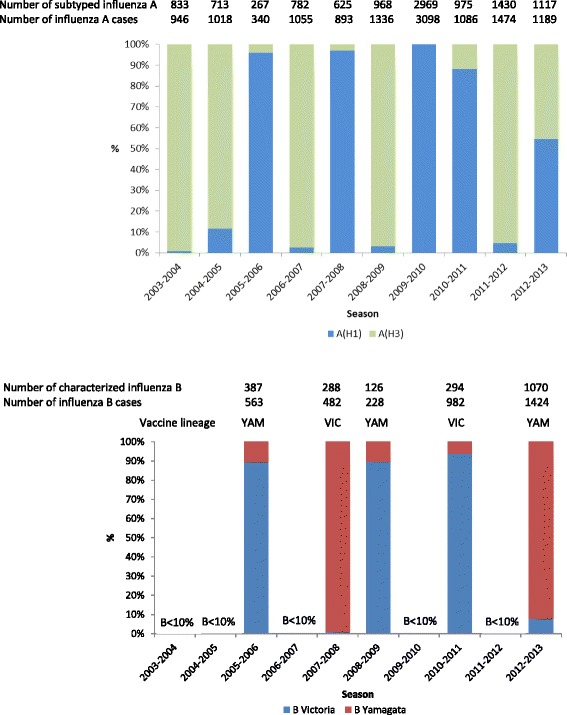
Percentages of subtyped influenza A cases by subtype and season (upper panel), and of characterized influenza B cases by lineage and season (lower panel; only for seasons when influenza B accounted for at least 10 % of all influenza cases) Source: GROG influenza sentinel surveillance network, France, 2003–2004 to 2012–2013

Overall, 85.9 % of influenza A viruses were subtyped: the subtypes A(H1) and A(H3) accounted for 51.6 % and 48.4 % of them, respectively. Influenza A(H1) and A(H3) were the most frequent influenza A subtype in four and, respectively, five seasons; the two virus subtypes co-circulated in 2012–2013 (Fig. 1). Influenza B accounted for at least 10 % of all influenza cases in five seasons, predominating in 2005–2006.

The lineage was characterized for 58.8 % of influenza B cases: the Yamagata and Victoria lineages accounted for 62.8 % and 37.2 % of them, respectively. In the five seasons where influenza B viruses circulated actively, B Yamagata and B Victoria were the most frequent lineage in two and, respectively, three seasons (Fig. 1). The influenza B lineage included in the vaccine and the dominant lineage were mismatched in three of these five seasons.

**Fig. 2:**
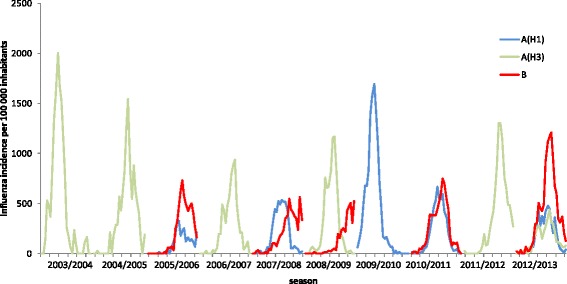
Estimated incidence (per 100,000 inhabitants) of medically-attended influenza A(H1), A(H3) and B, by season and week of onset of symptoms (weeks 40–15). Data were only shown for virus types and subtypes that accounted for at least 10 % of all influenza cases in that season Source: GROG influenza sentinel surveillance network, France, 2003–2004 to 2012–2013

The average estimated weekly incidence of medically-attended influenza during the study period was 191 per 100,000 inhabitants for influenza virus subtype A(H3), 58 per 100,000 inhabitants for the seasonal A(H1), 203 per 100,000 inhabitants for the pandemic A(H1) viruses, and 127 per 100,000 inhabitants for influenza B (yearly average and median values are available in Additional file 2: Table S1. Influenza A(H3) viruses were responsible of three of the five highest peaks of estimated weekly influenza incidence, including the highest estimated value (2,004 influenza cases per 100,000 inhabitants in week 49 of 2003) (Fig. 2). Pandemic influenza A(H1) viruses caused the second highest peak during the study period: 1,695 influenza cases per 100,000 inhabitants in week 49 of 2009. The highest peak of weekly estimated incidence of influenza B was 1,210 per 100,000 inhabitants in week 7 of 2013. The epidemics caused by influenza B viruses seemed to be still ongoing when surveillance was discontinued (in week 15) in 2007–2008 and 2008–2009 (Fig. 2).

The influenza B epidemics tended to take place a few weeks later than the influenza A epidemics in the five seasons where both virus types accounted for at least 10 % of all reported influenza cases (Table 2). In particular, influenza B peaked on average 3.8 weeks later than influenza A during such seasons. Instead, there were no differences between influenza A and B viruses in the average duration of the periods of intense circulation.

**Table 2:** Periods of active A(H1), A(H3) and B influenza virus circulation. Data were only shown for virus types and subtypes that accounted for at least 10 % of all influenza cases in that season

Season	Period of intense influenza activity ^(a)^			Peak week ^(b)^		
	A(H1)	A(H3)	B	A(H1)	A(H3)	B
2003–2004	-	43–51	-	-	47	
2004–2005	-	1–11	-	-	5	
2005–2006	2–13	-	3–13	5	-	7
2006–2007	-	52–9	-	-	5	-
2007–2008	51–8	-	2–14	4	-	8
2008–2009	-	50–6	5–15	-	3	11
2009–2010	43–52	-	-	48	-	-
2010–2011	50–7	-	49–8	2	-	5
2011–2012	-	3–13	-	-	9	-
2012–2013	50–10	50–12	52–13	4	6	7

Source: GROG influenza sentinel surveillance network, France, 2003–2004 to 2012–2013

^(a)^Defined as the shortest period that included the peak and at least 90 % of all influenza cases caused by that virus type/subtype that occurred during that season

^(b)^Defined as the week with the highest number of reported influenza cases of that type/subtype

## Discussion

We presented data on the circulation of influenza viruses in France from 2003–2004 to 2012–2013 based on information collected within a countrywide, community based sentinel influenza surveillance network. While influenza A viruses circulated all seasons, influenza B viruses circulated (i.e., accounted for at least 10 % of all influenza cases, according to the definition that we used throughout this paper) in half of the seasons. Influenza B accounted for around one fourth (23.7 %) of all influenza cases in France from 2003–2004 to 2012–2013 and caused more than 50 % of all influenza cases in two of ten seasons (2005–2006 and 2012–2013). No such historical data have been published in France but these results are in line with data of comparable quality from other European countries [12], for the totality of Europe [13–17], and for countries outside Europe as well [18, 19], despite differences in how the national influenza surveillance systems are organized in different countries.

The influenza virus A(H3) subtype circulated more often and was responsible for epidemics that were more intense (i.e., with a higher incidence peak) than those caused by the A(H1) subtype. This was certainly true before the pandemic of 2009–2010; our data on seasons 2011–2012 and 2012–2013 seem to confirm this pattern, although there is a need to extend the surveillance for a few years to be assured that the situation is now similar to what it was before the pandemic. Again, this is consistent with data from other countries, both in Europe [13–17] and outside [18–21].

When influenza B viruses circulated, one lineage was usually responsible for the great majority of all influenza B cases in the season. The dominant circulating B virus and the B virus in the vaccine were mismatched in three out of five seasons. Predictions about which B lineage will dominate in an upcoming season have largely failed worldwide during recent years [2]. Our data thus suggest that a quadrivalent influenza vaccine, including the two B lineages, may probably represents a useful tool to reduce the burden of influenza B, which in some seasons has a high incidence with significant public health and economic impact [18, 22–24].

We showed that influenza B peaks on average 3.8 weeks later than influenza A when both virus types circulate in the same season. Usually, the protection conferred by the vaccine against influenza covers the whole influenza season but it has been reported that this protection may decline quite rapidly (3–4 months after vaccination) within a given season especially in the elderly [25–27]. This may have important public health implications in seasons where both virus types circulate and cause epidemics that are spaced from one another by about one month, as in 2008–2009. In such seasons, it is possible that vaccinated frail people have poor residual protection against influenza B viruses, especially when B virus circulation peaks from late February onwards.

A major strength of our study is the fact that it relies on data that originate from a representative network of sentinel primary care practitioners spread over the whole French territory and therefore giving a strong picture of the circulation of influenza viruses in the general population. Our study has some limitations as well. The decision on which ARI patients to swab was left to the practitioners, which may have introduced some bias in the study. Our data include only three seasons after the season 2009–2010, which makes it difficult to establish with certainty whether the circulation pattern of influenza viruses has changed after the pandemic. The transition to the exclusive use of RT-PCR since the 2009–2010 season may have increased the influenza positivity rate of ARI patients of post- compared to pre-pandemic seasons. In some seasons, the influenza circulation was still ongoing when surveillance was discontinued in week 15. This occurred more often for influenza B viruses, as circulation of the latter tends to take place a few weeks after that of influenza A viruses. For instance, the influenza B epidemic was still ongoing on week 15 in seasons 2007–2008 and 2008–2009. Looking at Fig. 2, one can even hypothesize that the peak of influenza B occurred after week 15 in 2008–2009. As a consequence, it is likely that the proportion of influenza cases caused by type B viruses is somewhat underestimated in our study. The surveillance scheme should be extended by at least four weeks to include late epidemics caused by influenza B viruses and have a more precise picture of the circulation of influenza in the country. Another limitation is the lack of information on the age distribution of influenza cases, overall and by virus type and subtype/lineage. Finally, as in all GP-based influenza surveillance systems, our results most likely under-estimate the actual incidence of influenza, as not all influenza patients seek care when having ARI symptoms.

## Conclusions

Influenza type B virus is responsible of a substantial share of all influenza cases, up to over 50 % in seasons where influenza A viruses circulate with low incidence. The ability to predict the dominant lineage has been poor during recent years, which led to frequent influenza B vaccine mismatches. In this context, in 2013, the WHO recommendations included a second influenza B strain allowing countries to decide to recommend a trivalent or a quadrivalent influenza vaccine, which is expected to reduce the number of influenza cases and influenza-related hospitalizations and deaths. The epidemiology of influenza is however constantly changing under the effect of various driving forces [28, 29]: this makes it critical that influenza surveillance systems are in place and collect reliable and timely data on the circulation of influenza viruses, to allow health policy-makers and planners to optimize the strategies for prevention and control of influenza. It is also a key to guide research efforts to develop and adapt well-tailored influenza vaccines capable of reducing the influenza burden of disease in all age groups.

## Additional files

Additional file 1:
**GROG network.** ARI and influenza incidence estimation. (DOC 92 kb) Additional file 2: Table S1. Weekly average (and median) of the estimated incidence (per 100,000 inhabitants) of medically-attended influenza. Data were only shown for virus type and subtypes that accounted for at least 1 % of all influenza cases in that season. Source: GROG influenza sentinel surveillance network, France, 2003–2004 to 2012–2013. (DOC 31 kb)

## Abbreviations

WHO: World Health Organization
GROG: Groupes Régionaux d’Observation de la Grippe
GP: General practitioner
ARI: Acute respiratory infections
NIC: National Influenza center
RT-PCR: Real-time reverse transcriptase polymerase chain reaction
