# Supplementary material for: Ten influenza seasons in France: distribution and timing of influenza A and B circulation, 2003–2013
Source: BMC Infect Dis. 2015 Aug 21;15:357. doi: 10.1186/s12879-015-1056-z (PMC4545988; doi:10.1186/s12879-015-1056-z)
Supplement: Additional file 1: — GROG network. ARI and influenza incidence estimation. (DOC 92 kb) [file 12879_2015_1056_MOESM1_ESM.doc]

## GROG network

## ARI and influenza incidence estimation

October 2014

**A/ARI Incidence**

As part of its participation in the European influenza surveillance system (EISN - European Influenza Surveillance Network), the GROG network implemented, from the 2001-2002 season, a method of calculating the incidence of acute respiratory infections (ARI) per 100,000 inhabitants.

## A/ Required data

**A1/** **Clinical data from GROG network**

Number of ARI[[1]](#footnote-2) by age group[[2]](#footnote-3), by function (GP[[3]](#footnote-4)/Ped[[4]](#footnote-5)), for each French metropolitan region and each week,

Number of GROG GPs participating[[5]](#footnote-6) by region and week,

Number of GROG paediatricians participating[[6]](#footnote-7)5 by region and week.

These data are provided by the GROG data base collecting data by region and week.

**A2/ Demographics data**

**Estimate number of liberal GPs and paediatricians by French region**

Number of liberal GPs and paediatricians by region are annually provided by the Directorate for Research, Studies, Evaluation and Statistics (Direction de la recherche, des études, de l’évaluation et des statistiques, DRESS) in a document entitled « Doctors - Estimates on dd/mm/yyyy ».

Number of GPs by region results from the table « Effectifs des généralistes par région et secteur d’activité ». We only used number of GPs working in a consulting-room.

Number of paediatricians by region results from the table « Effectifs des médecins par région et secteur d’activité ». We only used number of paediatricians working in a consulting-room.

These data are updated each season.

For example, for 2001-202 season, we used data from a document entitled « Les médecins – Estimations au 1^er^ janvier 2001 » published in October 2001.

For 2007-2008 season, we used data from a document entitled « Les médecins – Estimations au 1^er^ janvier 2007 » published in September 2007.

For 2012-2013 season, we used data from a document entitled « Les médecins – Estimations au 1^er^ janvier 2008 » published in February 2012.

**Estimates of French population by age group and region**

Estimates of French population by age group and region are provided by the France’s National Institute for Statistics and Economic Studies (Institut National de la Statistique et des Etudes Economiques, INSEE) in the document « Estimations localisées de population au 1^er^ janvier par région, sexe et âge ».

For example, for 2012-2013 season, we used data concerning 2010 (the most recent data available).

**Summary table of needed data**

Sources variables abbreviation

**GROG clinical data**

GROG GP 0-4 years ARI Number 0-4 ARI GP

5-14 years ARI Number 5-14 ARI GP

15-64 years ARI Number 15-64 ARI GP

65 years or more ARI Number 65+ ARI GP

Number of GROG GP participating GROG GP

GROG paediatricians 0-4 years ARI Number 0-4 ARI Ped

5-14 years ARI Number 5-14 ARI Ped

15-64 years ARI Number 15-64 ARI Ped

Number of GROG paediatricians participating GROG Ped

**Demographics data**

DRESS Number of GPs nGP

Number of paediatricians nPed

INSEE Number of 0-4 years old inhabitants Pop 0-4

Number of 5-14 years old inhabitants Pop 5-14

Number of 15-64 years old inhabitants Pop 15-64

Number of 64 years old or more inhabitants Pop 65+

##### B/ Calculation assumptions

The ARI incidence (per 100,000) calculation is based on two assumptions:

- the sample of GROG GPs and paediatricians is representative of all colleagues in the region,
- GROG GPs and paediatricians go on vacation at the same time and in the same proportion as their colleagues.

### C/ Weekly ARI incidence calculation for a specific region, week and age group

Classically, for a specific week w, ARI incidence is calculated as:

ARI number*(w)* * French GP number

**ARI Incidence(w*)*** = ---------------------------------------------------------------------------------

number of GROG GP participating*(w)* * French population

ARI number(w)

**ARI Incidence(w)** = --------------------------------------------------------------------------------------------------

(number of GROG GP participating(w) * French population)/ French GP number

Thus, the classic denominator is:

number of GROG GP participating(w) * French population

**Denominator*(w)*** = ----------------------------------------------------------------------------

French GP number

**C1/** **Denominator calculation**

In our calculation, the denominator is built differently to take into account two types of doctors (general practitioners and paediatricians) while balancing their number relative to the number of IRA declared by each of these two categories of doctors.

Thus, the used denominator is:

**French population * (ARI GP*(w)* + IRA Ped*(w)*)**

**Denpop*(w)* = ---------------------------------------------------------------------------------------------------------------------------------------**

**French ped number French GP number**

**---------------------------------------------------- * ARI Ped*(w* + ------------------------------------- * ARI GP*(w)***

**number of GROG ped participating(w) number of GROG GP participating(w)**

The denominator is calculated weekly, for each region and each of the four age groups.

The calculation of the denominator for the age group A, in the region R, for the week W is:

**Pop A_R * (ARI GP_A_R_W + ARI Ped_A_R_W)**

**Denpop_A_R_W = ---------------------------------------------------------------------------------------------------------------------------------**

**nPed_R nGP_R**

**----------------------------------- * ARI Ped_A_R_W + ------------------------------------ * IRA GP_A_R_W**

**GROG Ped_R_W GROG GP_R_W**

where

Pop A_R Number of inhabitants belonging to age group A in region R

ARI GP_A_R_s Number of ARI belonging to age group A by GROG GP in region R during week w

ARI Ped_A_R_s Number of ARI belonging to age group A by GROG paediatricians in region R during week w

GROG GP_R_s Number of GROG GP participating in region R during week w

GROG Ped_R_s Number of GROG paediatricians participating in region R during week w

nGP_R Number of French GP in region R

nPed_R Number of French paediatricians in region R

**C2/** **Incidence Calculation**

**ARI incidence in region R belonging to age group A et during week w** is calculated as:

ARI GP_A_R_W + ARI Ped_A_R_W

**Incidence_A_R_W** = --------------------------------------------------------------

Denpop_A_R_W

**ARI incidence belonging to age group A et during week w** is calculated by adding all regions:

ARI GP_A_R_W + ARI Ped_A_R_W

**Incidence_A_W** = -------------------------------------------------------------------------

Denpop_A_R_W

**ARI incidence during week w** is calculated by adding all age groups:

ARI GP_A_R_W + ARI Ped_A_R_W

**Incidence_W** = -------------------------------------------------------------------------

Denpop_A_R_W

**ARI incidence during week w (per 100.000 inhabitants)** is calculated as:

ARI GP_A_R_W + ARI Ped_A_R_W

**Incidence_W_100000_** = ------------------------------------------------------------------------- * 100 000

Denpop_A_R_W

##### D/ Restrictions

###### **D1/ Reliability parameters**

The least reliable parameters are the regional numbers of GPs and, to a lesser extent, the number of paediatricians in the region. Indeed, the data provided by ADELI put together in one category of general practitioners those who actually see patients with ARI and those whose practice does not allow the reception of such patients (sports doctors, osteopathic physicians, acupuncturists, etc.). The use of other files (College of Physicians, etc.) is no more satisfying because these records are under the general designation, physicians whose activity has little to do with this specialty (epidemiologists for example), or even retired physicians.

###### **D2/ Case in which no IRA cases are reported**

If in a region and for a given week, paediatricians reported no ARI, the denominator no longer takes into account the number of GROG paediatrician participating and the number of paediatrician in the region. This therefore leads to an overestimation of the incidence of ARI.

If in a region and for a given week, paediatricians and general practitioners reported no ARI, the denominator is no longer calculable and by default takes the value 0. This situation, quite rare, also leads to an overestimation of incidence of ARI.

# ARI incidence during week w is calculated by adding all age groups:

ARI GP_A_R_W + ARI Ped_A_R_W

**Incidence_W** = --------------------------------------------------------------------

Denpop_A_R_W

In this way, the incidence of all age group during week w equals to the total of the 4 incidences by age group.

**B/ Influenza Incidence**

##### Influenza positive rate calculation

Influenza positive rate is calculated each week and for each influenza type and subtype as follows:

Nb Positive swabs for A(H3)

Influenza_posite_rate_W_H3 = -------------------------------------------------------

Nb total swabs for week W

Nb Positive swabs for A(H1)

Influenza_posite_rate_W_H1 = -------------------------------------------------------

Nb total swabs for week W

Nb Positive swabs for B

Influenza_posite_rate_W_B = -------------------------------------------------------

Nb total swabs for week W

Weekly influenza incidence is calculated as follows:

Incidence_Influenza_H3 = Incidence_AR_W * Influenza_posite_rate_W_H3

Incidence_Influenza_H1 = Incidence_AR_W * Influenza_posite_rate_W_H1

Incidence_Influenza_B = Incidence_AR_W * Influenza_posite_rate_W_B

1. ARI: sudden onset of at least one respiratory signs (cough, sore throat, shortness of breath, coryza...) AND at least one systemic sign suggestive of an acute infectious disease (fever, fatigue, headache, malaise...) [↑](#footnote-ref-2)
2. GROG age groups are: 0-4 years, 5-14 years, 15-64 years, 65 years or more. [↑](#footnote-ref-3)
3. GP: general practitioner [↑](#footnote-ref-4)
4. Ped : paediatrician [↑](#footnote-ref-5)
5. doctors participating : is considered as participating, GP or paediatrician having worked and sent his activity data to his regional coordination. [↑](#footnote-ref-6)
6. ^5^ [↑](#footnote-ref-7)
