# Supplementary material for: Ten influenza seasons in France: distribution and timing of influenza A and B circulation, 2003–2013
Source: BMC Infect Dis. 2015 Aug 21;15:357. doi: 10.1186/s12879-015-1056-z (PMC4545988; doi:10.1186/s12879-015-1056-z)
Supplement: Additional file 2: Table S1. — Weekly average (and median) of the estimated incidence (per 100,000 inhabitants) of medically-attended influenza. Data were only shown for virus type and subtypes that accounted for at least 1 % of all influenza cases in that season. Source: GROG influenza sentinel surveillance network, France, 2003–2004 to 2012–2013. (DOC 31 kb) [file 12879_2015_1056_MOESM2_ESM.doc]

**Supplementary table**. Weekly average (and median) of the estimated incidence (per 100,000 inhabitants) of medically-attended influenza. Data were only shown for virus type and subtypes that accounted for at least 1% of all influenza cases in that season.

Source: GROG influenza sentinel surveillance network, France, 2003-2004 to 2012-2013.

| **Season** | **B** | **A(H3)** | **A(H1)** |
| --- | --- | --- | --- |
| **2003-2004** | - | 482 (169) | - |
| **2004-2005** | - | 356 (209) | - |
| **2005-2006** | 210 (62) | - | 96 (60) |
| **2006-2007** | - | 245 (155) | - |
| **2007-2008** | 183 (116) | - | 179 (54) |
| **2008-2009** | 125 (25) | 291 (67) | - |
| **2009-2010** | - | - | 422 (163) |
| **2010-2011** | 241 (116) | - | 197 (61) |
| **2011-2012** | - | 352 (220) | - |
| **2012-2013** | 385 (309) | 144 (113) | 169 (85) |
